# Association Between Diabetes, Chronic Kidney Disease, and Outcomes in People With Heart Failure From Asia

**DOI:** 10.1016/j.jacasi.2023.03.005

**Published:** 2023-05-02

**Authors:** Claire A. Lawson, Wan Ting Tay, Lizelle Bernhardt, A. Mark Richards, Francesco Zaccardi, Jasper Tromp, Tiew-Hwa Katherine Teng, Chung-Lieh Hung, Chanchal Chandramouli, Gurpreet Singh Wander, Wouter Ouwerkerk, Sam Seidu, Kamlesh Khunti, Carolyn S.P. Lam, ASIAN-HF Investigators

**Affiliations:** a Department of Cardiovascular Sciences, University of Leicester, Leicester, United Kingdom; b Leicester Real World Evidence Unit, Leicester, United Kingdom; c National Heart Centre Singapore, Singapore, Singapore; d Yong Loo Lin School of Medicine, National University of Singapore, Singapore; e Christchurch Heart Institute, University of Otago, Dunedin, New Zealand; f National University Heart Centre, Singapore; g Diabetes Research Centre, Leicester, United Kingdom; h National Institute for Health Research Applied Research Collaboration–East Midlands, University of Leicester, Leicester, United Kingdom; i Saw Swee Hock School of Public Health, National University of Singapore and National University Health System, Singapore; j Duke-National University of Singapore Medical School, Singapore, Singapore; k School of Allied Health, University of Western Australia, Perth, Western Australia, Australia; l Department of Cardiology, Mackay Memorial Hospital, Taipei, Taiwan; m Department of Cardiology, Hero Heart Institute, Dayanand Medical College and Hospital, Ludhiana, India; n Department of Dermatology, University of Amsterdam Medical Center, Amsterdam, the Netherlands; o Department of Cardiology, University of Groningen, Groningen, the Netherlands

**Keywords:** chronic kidney disease, comorbidity, diabetes, epidemiology, heart failure, outcomes

## Abstract

**Background:**

Diabetes mellitus (DM), chronic kidney disease (CKD), and heart failure (HF) are pathophysiologically linked and increasing in prevalence in Asian populations, but little is known about the interplay of DM and CKD on outcomes in HF.

**Objectives:**

This study sought to investigate outcomes in patients with heart failure with preserved ejection fraction (HFpEF) vs heart failure with reduced ejection fraction (HFrEF) in relation to the presence of DM and CKD.

**Methods:**

Using the multinational ASIAN-HF registry, we investigated associations between DM only, CKD only, and DM+CKD with: 1) composite of 1-year mortality or HF hospitalization; and 2) Kansas City Cardiomyopathy Questionnaire scores, according to HF subtype.

**Results:**

In 5,239 patients with HF (74.6% HFrEF, 25.4% HFpEF; mean age 63 years; 29.1% female), 1,107 (21.1%) had DM only, 1,087 (20.7%) had CKD only, and 1,400 (26.7%) had DM+CKD. Compared with patients without DM nor CKD, DM+CKD was associated with 1-year all-cause mortality or HF hospitalization in HFrEF (adjusted HR: 2.07; 95% CI: 1.68-2.55) and HFpEF (HR: 2.37; 95% CI: 1.40-4.02). In HFrEF, DM only and CKD only were associated with 1-year all-cause mortality or HF hospitalization (both HRs: 1.43; 95% CI: 1.14-1.80), while in HFpEF, CKD only (HR: 2.54; 95% CI: 1.46-4.41) but not DM only (HR: 1.01; 95% CI: 0.52-1.95) was associated with increased risk (interaction *P <* 0.01). Adjusted Kansas City Cardiomyopathy Questionnaire scores were lower in patients with DM+CKD (HFrEF: mean 60.50, SEM 0.77, HFpEF: mean 70.10, SEM 1.06; *P <* 0.001) than with no DM or CKD (HFrEF: mean 66.00, SEM 0.65; and HFpEF: mean 75.80, SEM 0.99).

**Conclusions:**

Combined DM and CKD adversely effected outcomes independently of HF subtype, with CKD a consistent predictor of worse outcomes. Strategies to prevent and treat DM and CKD in HF are urgently required.

Heart failure (HF), diabetes mellitus (DM), and chronic kidney disease (CKD) are major pandemics of the 21st century. Increasing incidence of obesity and hypertension, alongside aging of the population, means that the prevalence of all 3 conditions is rising, with the most rapid increase in rates in developing countries.^1^ In Asia, DM develops at a much younger age and at a lower mean body mass index (BMI) than in the United States.^2^ The prevalence of HF in some parts of Asia is 2- to 3-fold that found in the United Kingdom and United States, presenting up to 20 years earlier,^3^ and nearly one-third of all cases of CKD are in China or India.^4^

HF is often clinically differentiated by ventricular ejection fraction, defined as heart failure with reduced ejection fraction (HFrEF) or heart failure with preserved ejection fraction (HFpEF). DM and CKD appear to play an important role in the pathogenesis of both HFpEF and HFrEF,^5^ albeit with potentially different pathophysiological mechanisms and associated risk factors. While DM^6^, ^7^, ^8^, ^9^ and CKD^10^, ^11^, ^12^, ^13^ individually worsen prognosis in HF, evidence by HF phenotype is limited and conflicting.^14^, ^15^, ^16^, ^17^, ^18^, ^19^, ^20^, ^21^, ^22^, ^23^, ^24^, ^25^ Furthermore, while outcomes for people with HF and DM have improved slightly over the past 2 decades, these improvements appear to be lost once CKD is present.^26^ Few studies, if any, have investigated the single and combined effects of these prevalent conditions by HF phenotype. This is important given that, until recently,^27^^,^^28^ no drug trials had demonstrated a reduction in cardiovascular death or hospitalization for HF in people with HFpEF. Additionally, there is now also an emergence of therapeutic agents that provide cardiorenal protective benefits for people with DM and CKD.^29^, ^30^, ^31^, ^32^

This study aimed to investigate the clinical correlates and outcomes of DM and CKD in patients with HFpEF and HFrEF in a multinational cohort in Asia and to disentangle the prognostic implications of DM, CKD, and combined DM and CKD.

## Methods

The data used in this study are not available to other researchers due to legal restrictions imposed by multinational jurisdictions.

### Population and setting

The ASIAN-HF registry is a multinational prospective observational registry of Asian patients, over 18 years of age, with symptomatic, stage C HF (presence of typical signs and symptoms of HF), and at least 1 episode of HF decompensation in the prior 6 months requiring hospitalization or treatment with intravenous diuretics at an outpatient clinic. This report included patients recruited from 42 medical centers covering a broad spectrum of medical, cardiology, and HF specialty units, in 10 regions (Taiwan, Hong Kong, India, Malaysia, Thailand, Singapore, Indonesia, Philippines, Japan, and Korea). Patients with HFrEF (ejection fraction <40%) were enrolled between October 1, 2012, and December 31, 2015, and patients with HFpEF (ejection fraction ≥50%) between September 9, 2013, and October 31, 2016, using uniform protocols and standardized procedures. Patients with severe valvular heart disease as the primary cause of HF or a life-threatening comorbidity with life-expectancy of <1 year were not included in the registry. Further details about the ASIAN-HF registry have been published previously.^33^

At recruitment all patients underwent 12-lead electrocardiography and standardized transthoracic echocardiography. We included all patients with HFrEF (ejection fraction <40%) and HFpEF (ejection fraction ≥50%). Within the registry, 99.5% of patients with HFpEF had echocardiographic evidence for diastolic dysfunction (E/e′ ≥13, E′ medial/lateral <9 ms, left atrial enlargement, or left ventricular hypertrophy).^34^ We excluded 1,394 (21.0%) patients who had missing information on DM or estimated glomerular filtration rate (eGFR).

### Exposures

We identified people with type 1 or type 2 DM by the presence of fasting plasma glucose ≥7 mmol/L, random plasma glucose ≥11.1 mmol/L, or glycated hemoglobin ≥6.5% or a self-reported history of DM and/or receiving antidiabetic therapy at baseline. CKD was defined by an eGFR <60 mL/min/1.73 m^2^, calculated using the Modification of Diet in Renal Disease formulary. Using the Kidney Disease: Improving Global Outcomes guidelines, CKD was further stratified by 4 severity groups, as follows: CKD-3a (eGFR 45-59 mL/min/1.73 m^2^, mild-to-moderate kidney disease), CKD-3b (eGFR 30-44 mL/min/1.73 m^2^, moderate to severe), CKD-4 (eGFR 15-29 mL/min/1.73 m^2^, severe), and CKD-5 (eGFR <15 mL/min/1.73 m^2^, kidney failure or dialysis).

HF patients were then categorized by the presence of DM and CKD, as follows: 1) DM 0, CKD 0 (reference group); 2) DM 1, CKD 0 (DM only); 3) DM 0, CKD 1 (CKD only); 4) DM 1, CKD 1 (DM+CKD).

### Covariates

We considered a range of clinically important variables, including socioeconomic factors (age, sex, ethnicity, geographical region [northeast, south, and southeast Asia], highest education level [none or primary, secondary, preuniversity, degree or higher], and household income), HF factors (inpatient or outpatient enrollment, NYHA functional class, heart rate and blood pressure), medications (angiotensin-converting enzyme inhibitors, angiotensin II receptor blockers, beta-blockers, mineralocorticoid receptor antagonists, diuretics, and statins), lifestyle factors (BMI, smoking, and alcohol intake), and comorbidities (coronary artery disease, atrial fibrillation, hypertension, stroke, peripheral arterial vascular disease, chronic respiratory disease, and anemia).

### Outcomes

The primary outcome of interest was a composite of all-cause mortality or hospitalization for HF at 1 year. Our secondary outcome was a composite of all-cause mortality or any hospitalization at 1 year. In addition, we estimated health-related quality of life (HRQoL) as assessed using the Kansas City Cardiomyopathy Questionnaire (KCCQ) at baseline registration. The KCCQ is a 23-item, self-administered questionnaire covering multiple domains in relation to health: physical function, symptoms, social function, self-efficacy, and knowledge. An overall summary score can be derived from each domain, with scores ranging from 0 (worse health possible) to 100 (best health possible).^35^ Non–English-speaking participants used certified versions of the KCCQ translated into their native languages. Outcomes were adjudicated by an independent committee.

### Statistical analysis

Baseline characteristic are first described by the presence or absence of DM, CKD, and combined DM+CKD and presented as number and percentage for categorical data and mean ± SD or median (IQR) for normally distributed and skewed continuous data, respectively. Groups were compared using analysis of variance, Wilcoxon rank sum test, or chi-square test, as appropriate (using alpha level of 0.05). Next, the sample was separated by HF subtype (HFrEF and HFpEF), and all characteristics were entered into a univariable logistic model followed by a multivariable model, to investigate independent associations with the presence of DM+CKD, compared with patients without DM or CKD. In the combined cohort including patients with HFrEF and HFpEF, an interaction term between HF subtype and each characteristic was also entered into the model, to assess effect modification by HF subtype. We also performed a sensitivity analysis to compare characteristics between patients with DM+CKD and patients with 1 or none of DM or CKD.

Unadjusted and adjusted associations of the exposure groups—DM only, CKD only and DM+CKD—compared with patients with no DM nor CKD (reference group), with the primary composite outcome of 1-year mortality or HF admission, were investigated using Cox models stratified by HF subtype; associations were reported as HRs with 95% CIs. An interaction term between the exposure groups and HF subtype was also entered into a single model to assess effect modification. We also performed a sensitivity analysis using the outcome of cardiovascular mortality or HF admission, and we used the same modeling approach to investigate our secondary outcome. To investigate HRQoL, we used linear regression to estimate the mean baseline KCCQ score for each exposure group and the SEM. We performed a sensitivity analysis removing 440 patients with type 1 DM. Supplementary analyses were also performed to assess the association between DM and CKD severity with the primary and secondary outcomes. Two-sided *P* values <0.05 were considered statistically significant. Statistical analyses were performed using Stata 15.0 (StataCorp).

### Ethics

Ethics approvals conforming to the Declaration of Helsinki were obtained from the relevant human ethics committees at all sites.

## Results

### Study population

There were 5,239 patients in the ASIAN-HF registry: the mean age was 63.1 ± 13.3 years, 1,524 (29.1%) were women, 1,394 (31.3%) had NYHA functional class III/IV, and 1,332 (25.4%) had HFpEF. Patients were generally younger, with less severe HF, but with higher prevalence of DM than found in other HF registries (Supplemental Table 1). Just under half of the patients had an inpatient enrollment (43.5%), and most patients were from high-income countries (62.0%) compared with low-income (27.1%) or middle-income (10.9%) countries. A total of 1,107 (21.1%) had DM only, 1,087 (20.7%) had CKD only, and 1,400 (26.7%) had DM+CKD (Table 1).

**Table 1:** Baseline Characteristics of Subjects by Presence of DM and CKD

	Missing	Overall (N = 5,239)	No DM, No CKD (n = 1,645)	DM Only (No CKD) (n = 1,107)	CKD Only (No DM) (n = 1,087)	DM+CKD (n = 1,400)	*P* Value
HFpEF	0 (0)	1,332 (25.4)	390 (23.7)	276 (24.9)	265 (24.4)	401 (28.6)	0.012
Age at baseline, y	0 (0)	63.1 ± 13.3	58.7 ± 14.5	61.1 ± 11.2	67.3 ± 13.5	66.7 ± 11.1	<0.001
Female	0 (0)	1,524 (29.1)	465 (28.3)	272 (24.6)	322 (29.6)	465 (33.2)	<0.001
Geographical region	0 (0)						<0.001
Northeast Asia		1,826 (34.9)	674 (41.0)	355 (32.1)	409 (37.6)	388 (27.7)	
South Asia		1,091 (20.8)	410 (24.9)	266 (24.0)	199 (18.3)	216 (15.4)	
Southeast Asia		2,322 (44.3)	561 (34.1)	486 (43.9)	479 (44.1)	796 (56.9)	
Regional income level	0 (0)						<0.001
Low		1,423 (27.1)	503 (30.6)	316 (28.5)	302 (27.8)	302 (21.6)	
Middle		569 (10.9)	156 (9.5)	116 (10.5)	128 (11.8)	169 (12.1)	
High		3,247 (62.0)	986 (59.9)	675 (61.0)	657 (60.4)	929 (66.4)	
Household income	830 (15.8)						<0.001
<$1,000		2,294 (52.0)	661 (46.1)	480 (51.7)	506 (54.1)	647 (58.2)	
$1,000-$2,999		884 (20.1)	319 (22.3)	184 (19.8)	173 (18.5)	208 (18.7)	
≥$3,000		503 (11.4)	214 (14.9)	102 (11.0)	101 (10.8)	86 (7.7)	
Decline to respond		728 (16.5)	239 (16.7)	163 (17.5)	155 (16.6)	171 (15.4)	
Highest education	830 (15.8)						<0.001
None or primary		1,411 (32.0)	391 (27.3)	266 (28.6)	325 (34.8)	429 (38.6)	
Secondary		1,370 (31.1)	455 (31.8)	301 (32.4)	270 (28.9)	344 (30.9)	
Preuniversity		612 (13.9)	219 (15.3)	149 (16.0)	124 (13.3)	120 (10.8)	
Degree or higher		860 (19.5)	317 (22.1)	186 (20.0)	174 (18.6)	183 (16.5)	
Decline to respond		156 (3.5)	51 (3.6)	27 (2.9)	42 (4.5)	36 (3.2)	
Ethnicity	0 (0)						<0.001
Chinese		1,792 (34.2)	518 (31.5)	368 (33.2)	369 (33.9)	537 (38.4)	
Indian		1,330 (25.4)	449 (27.3)	350 (31.6)	215 (19.8)	316 (22.6)	
Malay		819 (15.6)	176 (10.7)	175 (15.8)	174 (16.0)	294 (21.0)	
Japanese/Korean		975 (18.6)	402 (24.4)	170 (15.4)	237 (21.8)	166 (11.9)	
Thai/Filipino/**o**ther		323 (6.2)	100 (6.1)	44 (4.0)	92 (8.5)	87 (6.2)	
Inpatient enrollment	0 (0)	2,281 (43.5)	581 (35.3)	463 (41.8)	495 (45.5)	742 (53.0)	<0.001
NYHA functional class III/IV	791 (15.1)	1,394 (31.3)	378 (27.1)	282 (30.2)	310 (34.0)	424 (35.2)	<0.001
LVEF at baseline	0 (0)	31 (23-50)	30.0 (23.0-39.0)	30.0 (23.0-39.9)	30.3 (23.0-39.0)	32.0 (25.0-53.0)	0.003
BMI, kg/m^2^	40 (0.8)	25.4 (5.6)	24.8 (5.8)	24.2 (4.9)	26.4 (5.6)	26.1 (5.6)	<0.001
Heart rate, beats/min	29 (0.6)	77 (68-88)	79.1 ± 16.2	80.3 ± 15.9	77.7 ± 16.6	78.6 ± 15.2	0.002
Systolic BP, mm Hg	29 (0.6)	120 (108-134)	119.2 ± 20.2	122.2 ± 20.8	120.7 ± 22.5	126.6 ± 22.1	<0.001
Diastolic BP, mm Hg	3 (0.1)	70 (62-80)	72.9 ± 13.0	72.9 ± 12.4	71.5 ± 13.5	71.0 ± 12.4	<0.001
Coronary artery disease	2 (0)	2,486 (47.5)	571 (34.8)	594 (53.7)	461 (42.4)	860 (61.5)	<0.001
Atrial fibrillation/flutter	2 (0)	1,151 (22.0)	345 (21.0)	187 (16.9)	309 (28.4)	310 (22.1)	<0.001
History of hypertension	1 (0)	3,193 (61.0)	699 (42.5)	758 (68.5)	630 (58.0)	1,106 (79.1)	<0.001
Prior stroke	4 (0.1)	422 (8.1)	90 (5.5)	93 (8.4)	90 (8.3)	149 (10.6)	<0.001
Peripheral arterial vascular disease	1 (0)	178 (3.4)	27 (1.6)	36 (3.3)	23 (2.1)	92 (6.6)	<0.001
Chronic respiratory disease	884 (16.9)	459 (8.8)	137 (8.3)	101 (9.1)	100 (9.2)	121 (8.6)	0.84
Anemia	2 (0)	2,033 (46.7)	394 (29.7)	362 (41.0)	462 (49.7)	815 (67.1)	<0.001
Smoking, ever vs never	3 (0.1)	2,155 (41.2)	691 (42.0)	495 (44.7)	437 (40.2)	532 (38.1)	0.007
Alcohol, ever vs never	58 (1.1)	1,369 (26.2)	481 (29.2)	323 (29.2)	260 (23.9)	305 (21.8)	<0.001
ACE inhibitor or ARB	58 (1.1)	3,861 (74.5)	1,323 (81.6)	907 (82.5)	736 (68.5)	895 (64.6)	<0.001
Beta-blocker	58 (1.1)	3,967 (76.6)	1,269 (78.3)	869 (79.1)	771 (71.7)	1,058 (76.3)	<0.001
MRA	58 (1.1)	2,430 (46.9)	862 (53.2)	587 (53.4)	468 (43.5)	513 (37.0)	<0.001
Diuretics	58 (1.1)	4,161 (80.3)	1,226 (75.6)	905 (82.3)	862 (80.2)	1,168 (84.3)	<0.001
Loop diuretics	58 (1.1)	3,647 (70.4)	1,065 (65.7)	785 (71.4)	756 (70.3)	1,041 (75.1)	<0.001
Statin	358 (6.8)	3,352 (64.6)	874 (53.7)	846 (76.8)	598 (55.6)	1,034 (74.4)	<0.001
Death in 1 y	791 (15.1)	484 (9.9)	99 (6.4)	71 (6.9)	114 (11.6)	200 (15.0)	<0.001

Values are n (%), median (IQR), or mean ± SD.

ACE = angiotensin-converting enzyme; ARB = angiotensin II receptor blocker; BMI = body mass index; BP = blood pressure; CKD = chronic kidney disease; DM = diabetes mellitus; HFpEF = heart failure with preserved ejection fraction; HFrEF = heart failure with reduced e ejection fraction; LVEF = left ventricular ejection fraction; MRA = mineralocorticoid receptor antagonist.

### Baseline associations of DM+CKD

Overall, compared with HF patients without, those with DM+ CKD were more likely to be older, be from Southeast Asia, be from a region with higher national income but with lower household income and personal education level, be of Chinese or Malay ethnicity, and have an inpatient enrollment with more severe HF (NYHA functional class III/IV) (Table 1). Patients with DM+CKD were also more likely to have a higher BMI and systolic blood pressure and have coronary artery disease, hypertension, peripheral arterial disease, and anemia (all *P* < 0.001). In the multivariable models stratified by HF subtype, common associations of DM+CKD across both HF subtypes were older age, residing within a high-income region, Malay ethnicity, higher BMI, higher systolic blood pressure, presence of anemia, and prescription of diuretics and statins (Table 2). In HFrEF, but not HFpEF, female sex (interaction *P =* 0.01), was independently associated with the presence of DM+CKD, while Japanese or Korean ethnicity was associated with reduced prevalence (Table 2). In the sensitivity analysis, comparing those with DM+CKD with those with 1 or none of DM or CKD, the associations were similar, with the addition of inpatient enrollment and presence of coronary artery disease and prior stroke reaching significance for associations with increased risk of DM+CKD in patients with HFrEF but not with HFpEF (Supplemental Table 2).

**Table 2:** Associations of Risk Factors With Presence of Combined DM and CKD in HFrEF and HFpEF

	HFrEF		HFpEF		Characteristic × HF Group (Adjusted *P*_interaction_)
	Unadjusted OR (95% CI)	Adjusted OR (95% CI)	Unadjusted OR (95% CI)	Adjusted OR (95% CI)	
Age at baseline	1.05 (1.05-1.06)	1.04 (1.03-1.05)	1.04 (1.02-1.05)	1.02 (1.00-1.04)	0.0660
Female	1.33 (1.09-1.61)	1.62 (1.22-2.16)	0.97 (0.74-1.29)	0.74 (0.46-1.18)	0.0104
Regional income level					0.7342
Low	1.00 (Ref)	1.00 (Ref)	1.00 (Ref)	1.00 (Ref)	
Middle	1.73 (1.30-2.29)	2.37 (1.39-4.04)	2.29 (1.13-4.63)	1.85 (0.46-7.44)	
High	1.57 (1.30-1.90)	2.61 (1.70-4.03)	1.29 (0.85-1.95)	4.34 (1.40-13.4)	
Ethnicity					0.9653
Chinese	1.00 (Ref)	1.00 (Ref)	1.00 (Ref)	1.00 (Ref)	
Indian	0.59 (0.47-0.73)	1.23 (0.77-1.98)	1.11 (0.74-1.65)	1.81 (0.62-5.31)	
Malay	1.29 (0.99-1.67)	2.47 (1.61-3.80)	3.74 (2.20-6.35)	2.57 (1.06-6.23)	
Japanese/Korean	0.36 (0.28-0.47)	0.49 (0.35-0.68)	0.49 (0.31-0.77)	0.62 (0.33-1.17)	
Thai/Filipino/**o**thers	0.72 (0.51-1.01)	1.72 (0.85-3.49)	2.11 (0.78-5.71)	3.57 (0.60-21.14)	
Inpatient enrollment	2.01 (1.69-2.38)	1.71 (1.34-2.17)	2.58 (1.91-3.48)	1.10 (0.68-1.78)	0.1265
NYHA functional class III/IV	1.41 (1.17-1.71)	1.40 (1.07-1.85)	1.87 (1.28-2.73)	1.31 (0.70-2.43)	0.1915
BMI, kg/m^2^	1.03 (1.02-1.05)	1.06 (1.04-1.09)	1.05 (1.02-1.08)	1.07 (1.03-1.11)	0.5175
Heart rate, beats/min	1.00 (0.99-1.00)	1.00 (0.99-1.01)	1.00 (0.99-1.02)	1.01 (0.99-1.03)	0.2052
Systolic BP, mm Hg	1.02 (1.01-1.02)	1.01 (1.00-1.02)	1.02 (1.01-1.02)	1.01 (1.00-1.03)	0.6587
Diastolic BP, mm Hg	0.99 (0.99-1.00)	1.00 (0.99-1.01)	0.97 (0.96-0.99)	0.99 (0.97-1.01)	0.0061
Coronary artery disease	3.55 (2.98-4.24)	2.07 (1.62-2.66)	2.69 (1.96-3.69)	1.80 (1.05-3.07)	0.9440
Atrial fibrillation/flutter	1.08 (0.87-1.33)	0.99 (0.73-1.35)	0.97 (0.72-1.33)	0.85 (0.51-1.44)	0.6038
History of hypertension	5.34 (4.45-6.42)	3.00 (2.35-3.83)	4.81 (3.28-7.06)	6.54 (3.43-12.48)	0.6279
Prior stroke	2.27 (1.64-3.13)	1.57 (1.03-2.38)	1.57 (0.95-2.61)	0.92 (0.39-2.19)	0.1951
Peripheral arterial vascular disease	4.22 (2.62-6.79)	1.99 (1.08-3.66)	4.82 (1.63-14.31)	4.87 (0.57-41.96)	0.3639
Chronic respiratory disease	1.00 (0.74-1.35)	0.75 (0.49-1.13)	1.13 (0.7-1.83)	1.59 (0.68-3.73)	0.6765
Anemia	4.08 (3.37-4.93)	2.89 (2.28-3.67)	7.86 (5.42-11.38)	5.55 (3.38-9.10)	0.1640
Smoking, ever vs never	0.92 (0.78-1.08)	0.94 (0.72-1.22)	0.76 (0.55-1.06)	0.45 (0.24-0.86)	0.3187
Alcohol, ever vs never	0.66 (0.54-0.79)	0.86 (0.66-1.12)	0.93 (0.63-1.39)	1.29 (0.66-2.50)	0.1149
ACE inhibitor or ARB	0.35 (0.28-0.43)	0.46 (0.35-0.61)	0.63 (0.47-0.85)	0.68 (0.41-1.13)	0.0704
Beta-blocker	0.83 (0.67-1.02)	1.02 (0.76-1.38)	1.16 (0.86-1.58)	1.31 (0.77-2.24)	0.2037
MRA	0.50 (0.42-0.59)	0.65 (0.51-0.82)	0.64 (0.44-0.91)	0.57 (0.32-1.01)	0.7214
Diuretics	1.62 (1.29-2.03)	1.68 (1.22-2.31)	2.23 (1.62-3.08)	1.97 (1.15-3.38)	0.7003
Statin	2.69 (2.24-3.23)	1.69 (1.31-2.20)	2.18 (1.62-2.92)	1.89 (1.15-3.11)	0.5979

All covariates were entered into the adjusted model. Associations are reported for the outcome; presence of combined DM and CKD vs no DM and no CKD.

HF = heart failure; HFrEF = heart failure with reduced ejection fraction; other abbreviations as in Table 1.

### Associations with all-cause mortality or HF hospitalization

Compared with patients with no DM nor CKD (reference group), DM+CKD was associated with a 2-fold increase in the rates of all-cause mortality or HF hospitalization at 1 year, in both patients with HFrEF (adjusted HR: 2.07; 95% CI: 1.68-2.55) and patients with HFpEF (adjusted HR: 2.37; 95% CI: 1.40-4.02) (Table 3). However, when comparing individual disease groups with the reference group, there were differences by HF subtype: in HFrEF, both the DM-only and CKD-only groups were associated with increased all-cause-mortality or HF admission, but in HFpEF, only the CKD-only group and not the DM-only group was associated with increased risk (interaction *P* = 0.01) (Central Illustration). Results were very similar for cardiovascular death and HF hospitalization in the sensitivity analysis (Supplemental Table 3). In both HF subtypes, there was an incremental increase in risk of the primary outcome (all-cause mortality or HF hospitalization) with CKD severity, reaching an HR of 2.72 (95% CI: 2.06-3.59) in HFrEF for DM+CKD-4 and an HR of 3.29 (95% CI: 1.56-6.94) in HFpEF for DM+CKD-5 (Supplemental Table 4).

**Table 3:** Hospitalization or Mortality in HFrEF and HFpEF

Comorbidity Group	HFrEF				HFpEF			
	No. at Risk	No. of Events	Unadjusted HR (95% CI)	Adjusted HR (95% CI)^a^	No. at Risk	No. of Events	Unadjusted HR (95% CI)	Adjusted HR (95% CI)^a^
All-cause deaths or heart failure hospitalization at 1 y								
No DM, no CKD	1,163	155 (13.3)	1.00 (Ref)	1.00 (Ref)	367	20 (5.5)	1.00 (Ref)	1.00 (Ref)
DM only	771	163 (21.1)	1.66 (1.33-2.07)	1.43 (1.14-1.80)	261	19 (7.3)	1.35 (0.72-2.53)	1.01 (0.52-1.95)
CKD only	735	170 (23.1)	1.84 (1.48-2.29)	1.43 (1.14-1.80)	248	45 (18.2)	3.62 (2.14-6.12)	2.54 (1.46-4.41)
DM+CKD	944	338 (35.8)	3.08 (2.55-3.73)	2.07 (1.68-2.55)	378	87 (23.0)	4.66 (2.87-7.58)	2.37 (1.40-4.02)
All-cause deaths or all-cause hospitalization at 1 y								
No DM, no CKD	1163	302 (26.0)	1.00 (Ref)	1.00 (Ref)	367	56 (15.3)	1.00 (Ref)	1.00 (Ref)
DM only	771	255 (33.1)	1.33 (1.13-1.57)	1.19 (1.00-1.41)	261	55 (21.1)	1.42 (0.98-2.07)	1.12 (0.76-1.66)
CKD only	735	261 (35.6)	1.44 (1.22-1.70)	1.16 (0.98-1.39)	248	79 (31.9)	2.34 (1.66-3.29)	1.71 (1.19-2.45)
DM+CKD	944	456 (48.4)	2.18 (1.88-2.52)	1.58 (1.34-1.85)	378	154 (40.7)	3.20 (2.36-4.35)	1.90 (1.36-2.66)

Values are n (%), unless otherwise indicated.

HRQoL = health-related quality of life; other abbreviations as in Tables 1 and 2.

a Adjusted for age, sex, ethnicity, enrollment type, regional income, systolic BP, heart rate, ejection fraction, coronary artery disease, chronic obstructive pulmonary disease, atrial fibrillation, peripheral arterial vascular disease, use of ACE inhibitors, ARBs, beta-blockers, and diuretics.

**Central Illustration:**
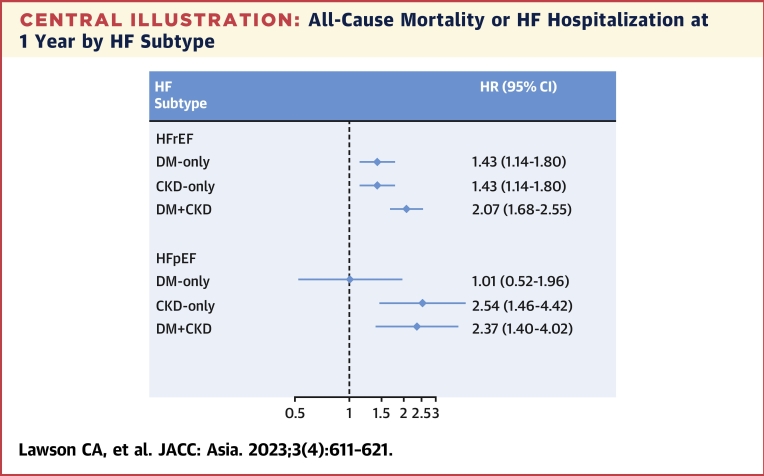
All-Cause Mortality or HF Hospitalization at 1 Year by HF Subtype Associations between the exposure groups, diabetes mellitus (DM) only, chronic kidney disease (CKD) only, and DM+CKD, compared with patients with no DM or CKD (reference group), with the primary composite outcome of 1-year mortality or heart failure (HF) admission. HFpEF = heart failure with preserved ejection fraction; HFrEF = heart failure with reduced ejection fraction.

### Associations with all-cause mortality or any hospitalization

Findings were similar for the composite of all-cause mortality or any hospitalization. Compared with patients with no DM or CKD, DM+CKD was associated with increase in the rates of all-cause mortality or any hospitalization at 1 year in HFrEF (HR: 1.58; 95% CI: 1.34-1.85) and HFpEF (HR 1.90; 95% CI: 1.36-2.66) (Tables 3 and 4), and there were differences by HF subtype. The association between individual diseases and increased rates were diminished in the HFrEF group, and in the HFpEF group, CKD but not DM was associated with higher rates.

**Table 4:** HRQoL in HFrEF and HFpEF

	HFrEF				HFpEF			
	Unadjusted		Adjusted^a^		Unadjusted		Adjusted^a^	
	Mean (SEM)	*P* Value	Mean (SEM)	*P* Value	Mean (SEM)	*P* Value	Mean (SEM)	*P* Value
KCCQ at baseline								
No DM, no CKD	67.20 (0.71)	Ref	66.00 (0.65)	Ref	78.90 (1.26)	Ref	75.80 (0.99)	Ref
DM only	65.20 (0.90)	0.081	65.30 (0.80)	0.485	74.80 (1.49)	0.036	72.70 (1.12)	0.037
CKD only	61.30 (0.88)	<0.001	61.60 (0.79)	<0.001	68.80 (1.61)	<0.001	71.70 (1.24)	0.012
DM+CKD	59.20 (0.84)	<0.001	60.50 (0.77)	<0.001	66.40 (1.34)	<0.001	70.10 (1.06)	<0.001

Abbreviations as in Tables 1 and 2.

a Adjusted for age, sex, ethnicity, enrollment type, regional income, systolic BP, heart rate, ejection fraction, coronary artery disease, chronic obstructive pulmonary disease, atrial fibrillation, peripheral arterial vascular disease, use of ACE inhibitors, ARBs, beta-blockers, and diuretics as well as for education.

### Health-related quality of life

**Figure 1:**
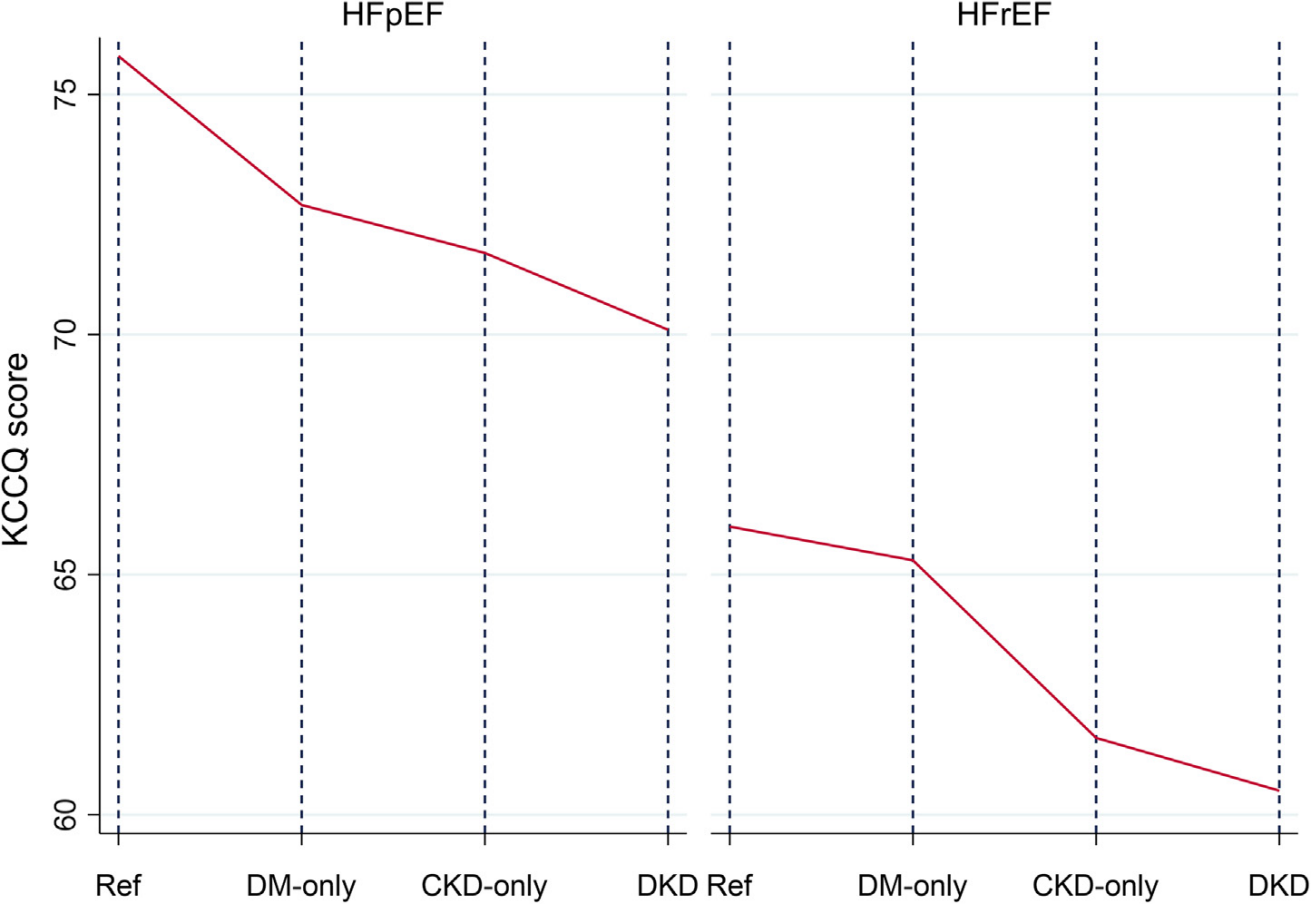
Baseline Health-Related Quality of Life in HFrEF and HFpEF With 95% CI Estimated health-related quality of life at baseline for each exposure group: no diabetes mellitus (DM) or chronic kidney disease (CKD) (reference group), DM only, CKD only, and DM+CKD (DKD), by heart failure subtype. HFpEF = heart failure with preserved ejection fraction; HFrEF = heart failure with reduced ejection fraction; KCCQ = Kansas City Cardiomyopathy Questionnaire.

By HF subtype, compared with the reference groups with no DM or CKD (HFrEF: mean 66.00, SEM 0.65; HFpEF: mean 75.80, SEM 0.99), the DM+CKD groups had the significantly lowest baseline KCCQ scores (HFrEF: mean 60.50, SEM 0.77, *P <* 0.001; HFpEF: mean 70.10, SEM 1.06, *P <* 0.001) (Table 4). In HFrEF, a significantly lower KCCQ score was observed for the CKD-only group and not the DM-only group, whereas in HFpEF, a significantly lower KCCQ score was observed in the CKD-only and DM-only groups (Tables 3 and 4, Figure 1). There was a pattern of reduced KCCQ score with the most severe CKD severity in those with and without DM and in both the HFrEF and HFpEF groups (Supplemental Table 4). All associations were similar after removing patients with HF and type 1 DM (Supplemental Table 5).

## Discussion

The combined presence of DM and CKD was significantly associated with more than 2-fold-higher risk of mortality or HF admission, in both HFrEF and HFpEF, but there were distinct differences in associations between HF subtypes and individual conditions. We show that both DM and CKD independently contributed to increased risk in HFrEF, but only CKD, and not DM, was associated with increased risk in HFpEF. CKD and increasing CKD severity was also the predominant driver of reduced HRQoL in both HF subtypes.

Existing data are conflicting, with few studies, if any, comparing HFrEF and HFpEF according to DM and CKD categorization. In prior trial populations, DM was associated with mortality and HF hospitalization in HFpEF.^15^^,^^36^^,^^37^ The PARAGON-HF (Prospective Comparison of Angiotensin Receptor–Neprilysin Inhibitor with Angiotensin-receptor Blockers Global Outcomes in HF with Preserved Ejection Fraction) trial only included patients with a known intolerance of renin-angiotensin system inhibitors, and renal function was not accounted for in the CHARM (Candesartan in Heart Failure Assessment of Reduction in Mortality and Morbidity) analyses. Given the consistent association between CKD and outcomes and the close association between DM and worsening renal function,^38^ this may partly explain these divergent reports. Compared with our Asian population, the patients in these prior studies were also older, with a higher proportion of patients with more severe HF and no adjustment was made for socioeconomic status. Our findings are consistent with prior evidence from Get With The Guidelines-Heart Failure cohort^18^; despite being an older population, patients had optimized treatments, with the majority prescribed guideline driven therapy. In another registry study set in 7 Middle Eastern countries^19^ with a similar age to our Asian population, DM had no association with mortality or admission. Both studies had similar high comorbidity rates to those found in our Asian population, and these findings may reflect less severe or shorter duration of DM, or the less perceptible influence of DM among the many different risk factors in these patients. Last, lack of current evidence-based treatment strategies for HFpEF may mean that DM may play less of an important role once HFpEF has developed.

CKD was a consistent predictor of poor outcomes in both HF subtypes. CKD may limit the use of some disease-modifying therapies in HF, such as those that inhibit the renin-angiotensin-aldosterone system. Furthermore, CKD in HF and DM may be underdiagnosed and undertreated,^39^ potentially leading to more severe CKD and acting as a counterbalance to any benefits gained through earlier cardiovascular disease prevention in people with DM. Our findings that CKD is associated with poor outcomes in HFrEF is consistent with prior evidence, but evidence in HFpEF has been inconsistent, with some studies finding no association with outcomes.^21^^,^^22^ Again, these studies included older and more severe HF patients than found in our registry, indicating that CKD may have a reduced relative effect in more severe HF populations. Our findings are consistent with those from the Cardiovascular Research Network PRESERVE study, which included ambulatory and in-hospital patients, similar to our cohort,^20^ and indicating a greater relative effect in patients with less severe HF.

In our patients, presence of both DM and CKD together was associated with the highest risk of mortality or hospitalization and the lowest HRQoL. These 3 conditions are linked by hemodynamic, neurohormonal, or inflammatory pathways and often exist together, each worsening the prognosis of the other 2 conditions.^5^ Compared with those without, patients with combined DM and CKD were more likely to be of Malay ethnicity and from a high-income country but with lower household income and education level, indicating the importance of a within-country economic divide. Higher prevalence of obesity and physical inactivity in high-income compared with low-income countries^40^ may disproportionality effect the poor and less educated groups. The lower prevalence of DM and CKD in Japan and Korea may reflect the lower economic divide in these regions, compared with the other high-income regions. While the higher prevalence of combined DM and CKD in Malay patients is not fully understood, it likely relates to multidimensional racial, economic, and health inequalities, requiring a culturally sensitive multidisciplinary approach that goes beyond lifestyle-centered decisions.41 Our findings of increased risk associated with combined DM and CKD is consistent with prior studies42^,^43 and highlights the need for therapeutics that improve outcomes in HF, while simultaneously improving health status. Improving physical function and reducing symptoms to improve health has become a major goal, recognized by international consensus on clinical outcomes in HF44 and clinical trials.45

The recent adoption of novel drug classes, including sodium-glucose cotransporter 2 inhibitors, alongside sacubitril/valsartan, into first-line guideline-driven medical therapy shows early promise.46 Trials have shown that, in addition to preventing HF hospitalizations in people with CKD,47-49 longer term use of sodium-glucose cotransporter 2 inhibitors has the potential to delay progression of CKD once HF has developed.^49^ Wider benefits in patients with HFrEF include increased survival, reduced hospitalizations and symptoms, and improved HRQoL.^32^^,^50 While the same benefits had not previously been shown for people with HFpEF, there have been some promising signs in women with HFpEF,51 and the EMPEROR-Preserved (Empagliflozin Outcome Trial in Patients with Chronic Heart Failure with Preserved Ejection Fraction) and DELIVER (Dapagliflozin Evaluation to Improve the LIVEs of Patients With PReserved Ejection Fraction Heart Failure) trials have now reported a significant benefit in people with HFpEF.^27^^,^^28^ These findings together indicate that irrespective of ejection fraction nephroprotective agents should be considered and that in people with HFpEF and DM, the focus on the use of agents that prevent renal deterioration may be more desirable than their glycemic reducing ability and should therefore not be withheld.

### Study Strengths and limitations

By using a multinational, multiethnic, prospective observational cohort, we were able to explore in detail the characteristics and outcomes of DM and CKD, which are growing in prevalence globally and at the highest rate in developing countries. We had access to echocardiographic data to explore differences by HF subtype and patient-reported health status to investigate HRQoL. We cannot rule out the potential for bias in the data collection across various centers or for participation bias within the ASIAN-HF registry, in which the patients that were willing and able to participate may differ in some way to the nonparticipants. However, standardized protocols were used with specific language translations, training, and monitoring, and participants were representative of single-country registers.52 We also acknowledge that by including prevalent cases of HF we could not assess the temporal relationship between baseline DM, CKD, and HF, meaning that there is potential for some residual confounding by disease duration and reverse causality, and further work is required to fully understand the interrelationships. Furthermore, we did not have complete data on albuminuria, and patients were studied before the widespread availability of newer antiglycemic agents (eg, sodium-glucose cotransporter 2 inhibitor).

## Conclusions

In a prospective registry of HF in Asia, the combination of DM and CKD posed a major health challenge, modulated by socioeconomic and ethnic differences. DM combined with CKD was associated with higher rates of most adverse outcomes independent of HF subtype, with increasingly severe renal dysfunction a consistent predictor of worse outcomes and reduced HRQoL. Strategies to optimize the prevention and treatment of DM and CKD in HF and to translate the recent promising sodium-glucose cotransporter 2 trial results into real-world patient benefit are urgently required.

Perspectives **COMPETENCY IN MEDICAL KNOWLEDGE:** People with DM, CKD, and HF are at high risk, requiring close consideration of comorbidity management.

**COMPETENCY IN PATIENT CARE AND PROCEDURAL SKILLS 1:** Irrespective of ejection fraction, nephroprotective agents should be considered.

**COMPETENCY IN PATIENT CARE AND PROCEDURAL SKILLS 2:** In people with HFpEF and DM, the focus on the use of agents that prevent renal deterioration may be more desirable than their glycemic reducing ability and should therefore not be withheld.

**TRANSLATIONAL OUTLOOK:** To better elucidate the interrelationships between DM, CKD, and HF, further work is needed to include consideration of temporality, disease duration, and severity.

## Funding Support and Author Disclosures

The ASIAN-HF study is supported by grants from Boston Scientific Investigator Sponsored Research Program, National Medical Research Council of Singapore, A∗STAR Biomedical Research Council ATTRaCT program, and Bayer. Dr Lawson is funded by the National Institute for Health Research (No. 30011). Dr Lam is supported by a Clinician Scientist Award from the National Medical Research Council of Singapore; has received research support from Bayer and Roche Diagnostics; has served as consultant or on the advisory board, steering committee, or executive committee for Actelion, Amgen, AnaCardio AB, Applied Therapeutics, AstraZeneca, Bayer, Boehringer Ingelheim, Boston Scientific, Cytokinetics, Darma Inc, EchoNous Inc, Impulse Dynamics, Ionis Pharmaceutical, Janssen Research & Development LLC, Medscape/WebMD Global LLC, Merck, Novartis, Novo Nordisk, Prosciento Inc, Radcliffe Group Ltd, Roche Diagnostics, Sanofi, and Us2.ai; and is co-founder & nonexecutive director of Us2.ai. Dr Zaccardi has received speaker fees from Napp Pharmaceuticals and Boehringer Ingelheim. Dr Tromp has received speaker fees from Daichii Sankyo and Roche Diagnostics; has received consultancy fees from Us2.ai; and holds a patent entitled “Automatic clinical workflow that recognizes and analyses 2D and doppler modality echocardiogram images for automated cardiac measurements and the diagnosis, prediction and prognosis of heart disease” unrelated to the present work. Dr Seidu reports receiving personal fees from Amgen, AstraZeneca, NAPP, Lilly, Merck Sharp & Dohme, Novartis, Novo Nordisk, Roche, Sanofi and Boehringer Ingelheim; has received grants from AstraZeneca, Sanofi, Servier and Janssen. All other authors have reported that they have no relationships relevant to the contents of this paper to disclose.
