# Supplementary material for: Association Between Diabetes, Chronic Kidney Disease, and Outcomes in People With Heart Failure From Asia
Source: JACC Asia. 2023 May 2;3(4):611–21. doi: 10.1016/j.jacasi.2023.03.005 (PMC10442874; doi:10.1016/j.jacasi.2023.03.005)
Supplement: Supplemental Appendix [file mmc1.docx]

## Supplemental Table 1: Characteristics in ASIAN-HF compared to other HF disease registries.

|  | Asian-HF | SwedeHF | REPORT-HF | Global-HF | HF audit | GWTG-HF |
| --- | --- | --- | --- | --- | --- | --- |
| Number included | 5,239 | 35,163 | 18,102 | 23,291 | 217392 | 423,333 |
| Age, median (IQR) or mean±SD | 63.1 (SD 13.3) | 74.0 66.0, 81.0) | 67 (57, 77) | 65 (SD 13) | 81 (72, 87) | 72+/114.6 |
| Female | 29.1 | 39.4 | 39 | 39 | 46 | 48.2 |
| HFpEF | 25 | 21 | 31 | 46 (EF ≥40%) | 46.5 | 43 |
| HFrEF | 75 | 55 | 50 | 54 | 53.5 | 43 |
| NYHA III/IV | 31 | 44 | 32 | 40 | 78 | NR |
| Diabetes | 48 | 24 | 37 | 31 | 30 | 45 |
| CKD | 47 | 53 | 20 | NR | NR | 25 |
| Beta blocker | 77 | 84 | 76 | 80 | 74 | 69 |
| ACEi or ARB | 75 | 80 | 70 | 77 | 83 | 48 |
| IHD | 48 | 51 | 48 | 38 | 39 | 50 |
| Atrial rhythm | 22 | 48 | 31 | 27 | 42 | 39 |
| IQR, Interquartile range; SD, standard deviation; HFpEF, heart failure with preserved ejection fraction ; HFrEF, heart failure with reduced ejection fraction; NYHA, New York Heart Association; CKD, chronic kidney disease ; ACEi, angiotensin-converting enzyme inhibitor; ARB, angiotensin receptor blocker; IHD, ischemic heart disease.   - Swede-HF^1^ (nationwide registry including patients from 70 of a total of 80 hospitals (both inpatients and outpatients) and from 100 of a total of 1000 outpatient primary care clinics in Sweden) - Report-HF^2^ (Post-discharge heart failure patients from 358 sites from 44 countries on six continents (Central and South America, Eastern Europe, Eastern Mediterranean region and Africa, North America, Southeast Asia, Western Europe & Western Pacific). - Global-HF^3^ (257 centers in 40 countries from 8 major geographic regions of the world (North America, Western Europe, Eastern Europe, the Middle East, South Asia, East Asia, Africa, and South America) - HF audit^4^ (England and Wales) (patients admitted to hospital for HF between 2012 and 2018) - GWTG-HF^5^ (adults hospitalized with HF from all regions and of various types across the United States). | | | | | | |

1. Johansson I, Dahlström U, Edner M, Näsman P, Rydén L, Norhammar A. Prognostic Implications of Type 2 Diabetes Mellitus in Ischemic and Nonischemic Heart Failure. J Am Coll Cardiol. 2016;68(13):1404-16.

2. Tromp J, Bamadhaj S, Cleland JGF, Angermann CE, Dahlstrom U, Ouwerkerk W, et al. Post-discharge prognosis of patients admitted to hospital for heart failure by world region, and national level of income and income disparity (REPORT-HF): a cohort study. The Lancet Global Health. 2020;8(3):e411-22.

3. Johansson I, Joseph P, Balasubramanian K, McMurray JJV, Lund LH, Ezekowitz JA, et al. Health-Related Quality of Life and Mortality in Heart Failure: The Global Congestive Heart Failure Study of 23 000 Patients From 40 Countries. Circulation. 2021;143(22):2129-42.

4. Gulea C, Zakeri R, Kallis C, Quint JK. Impact of COPD and asthma on in-hospital mortality and management of patients with heart failure in England and Wales: an observational analysis. BMJ Open. 2022;12(6):e059122.

5. Cunningham LC, Fonarow GC, Yancy CW, Sheng S, Matsouaka RA, DeVore AD, et al. Regional Variations in Heart Failure Quality and Outcomes: Get With The Guidelines-Heart Failure Registry. J Am Heart Assoc. 2021 Apr 6;10(7):e018696.

| Supplemental Table 2: Sensitivity analysis; Associations of risk factors with presence of combined DM and CKD (vs either or none of DM or CKD) in HFrEF and HFpEF | | | | | | | |
| --- | --- | --- | --- | --- | --- | --- | --- |
|  | **HFrEF** | |  | **HFpEF** | |  | **Characteristic x HF group** |
|  | **Unadjusted OR (95% CI)** | **Adjusted OR (95% CI)** |  | **Unadjusted OR (95% CI)** | **Adjusted OR (95% CI)** |  | **Adjusted**  **p-_interaction_** |
| Age at baseline, years | 1.03 (1.03-1.04) | 1.02 (1.02-1.03) |  | 1.02 (1.01-1.03) | 1.01 (0.99-1.02) |  | 0.0145 |
| Women | 1.38 (1.16-1.63) | 1.63 (1.32-2.03) |  | 1.03 (0.82-1.31) | 0.92 (0.65-1.29) |  | 0.0107 |
| Regional income level |  |  |  |  |  |  | 0.9255 |
| Low | 1.00 (Ref) | 1.00 (Ref) |  | 1.00 (Ref) | 1.00 (Ref) |  |  |
| Middle | 1.54 (1.21-1.95) | 1.81 (1.23-2.68) |  | 1.69 (0.97-2.96) | 1.02 (0.35-2.97) |  |  |
| High | 1.50 (1.27-1.77) | 2.05 (1.51-2.78) |  | 1.20 (0.84-1.72) | 2.04 (0.90-4.62) |  |  |
| Ethnicity |  |  |  |  |  |  | 0.6567 |
| Chinese | 1.00 (Ref) | 1.00 (Ref) |  | 1.00 (Ref) | 1.00 (Ref) |  |  |
| Indian | 0.64 (0.53-0.78) | 1.06 (0.75-1.48) |  | 1.05 (0.75-1.46) | 1.00 (0.50-2.02) |  |  |
| Malay | 1.08 (0.87-1.33) | 1.54 (1.15-2.06) |  | 2.54 (1.77-3.66) | 1.64 (0.94-2.88) |  |  |
| Japanese/Korean | 0.43 (0.34-0.54) | 0.56 (0.43-0.73) |  | 0.65 (0.43-0.99) | 0.80 (0.47-1.36) |  |  |
| Thai/Filipino/Others | 0.74 (0.55-1.00) | 1.22 (0.74-2.02) |  | 2.03 (0.94-4.41) | 2.18 (0.59-8.10) |  |  |
| Inpatient enrolment | 1.68 (1.45-1.94) | 1.49 (1.24-1.80) |  | 1.87 (1.47-2.37) | 0.94 (0.66-1.34) |  | 0.0081 |
| NYHA Class III/IV | 1.27 (1.08-1.49) | 1.23 (0.99-1.52) |  | 1.36 (1.02-1.82) | 1.08 (0.71-1.63) |  | 0.1672 |
| BMI, kg/m^2^ | 1.02 (1.01-1.04) | 1.04 (1.03-1.06) |  | 1.04 (1.01-1.06) | 1.06 (1.02-1.09) |  | 0.4345 |
| Heart rate, bpm | 1.00 (0.99-1.00) | 1.00 (1.00-1.01) |  | 1.00 (0.99-1.01) | 1.01 (0.99-1.02) |  | 0.6882 |
| Systolic BP, mmHg | 1.01 (1.01-1.02) | 1.01 (1.00-1.01) |  | 1.01 (1.01-1.02) | 1.01 (1.01-1.02) |  | 0.3328 |
| Diastolic BP, mmHg | 0.99 (0.99-1.00) | 1.00 (0.99-1.01) |  | 0.98 (0.97-0.99) | 0.99 (0.97-1.00) |  | 0.2635 |
| Coronary artery disease | 2.54 (2.18-2.96) | 1.87 (1.54-2.27) |  | 1.90 (1.48-2.42) | 1.14 (0.79-1.64) |  | 0.0469 |
| Atrial fibrillation/flutter | 1.01 (0.84-1.21) | 0.93 (0.74-1.18) |  | 0.95 (0.73-1.23) | 0.97 (0.67-1.42) |  | 0.8582 |
| History of hypertension | 3.20 (2.73-3.76) | 1.90 (1.56-2.31) |  | 3.05 (2.14-4.35) | 3.62 (2.15-6.10) |  | 0.1285 |
| Prior stroke | 1.65 (1.29-2.11) | 1.39 (1.03-1.87) |  | 1.32 (0.89-1.96) | 0.67 (0.37-1.21) |  | 0.0184 |
| Peripheral arterial vascular disease | 2.86 (2.06-3.97) | 1.80 (1.21-2.66) |  | 5.77 (2.5-13.29) | 6.89 (1.81-26.23) |  | 0.0536 |
| Chronic respiratory disease | 0.96 (0.74-1.24) | 0.82 (0.59-1.12) |  | 1.01 (0.68-1.49) | 0.93 (0.52-1.66) |  | 0.9421 |
| Anemia | 2.85 (2.43-3.34) | 2.23 (1.86-2.68) |  | 4.38 (3.22-5.94) | 4.16 (2.84-6.10) |  | 0.0617 |
| Smoking, ever vs never | 0.89 (0.77-1.03) | 0.88 (0.72-1.08) |  | 0.78 (0.59-1.04) | 0.50 (0.32-0.79) |  | 0.3891 |
| Alcohol, ever vs never | 0.73 (0.62-0.85) | 0.96 (0.78-1.18) |  | 0.85 (0.61-1.19) | 0.90 (0.55-1.46) |  | 0.3097 |
| ACEi or ARB | 0.45 (0.38-0.53) | 0.61 (0.50-0.75) |  | 0.71 (0.55-0.91) | 0.64 (0.44-0.92) |  | 0.3720 |
| Beta-blocker | 0.92 (0.77-1.09) | 1.01 (0.80-1.27) |  | 1.23 (0.95-1.60) | 1.41 (0.96-2.06) |  | 0.0709 |
| MRA | 0.56 (0.49-0.65) | 0.72 (0.60-0.87) |  | 0.68 (0.50-0.93) | 0.62 (0.39-0.96) |  | 0.9136 |
| Diuretics | 1.31 (1.07-1.61) | 1.36 (1.05-1.76) |  | 1.89 (1.43-2.50) | 1.85 (1.23-2.80) |  | 0.2852 |
| Statin | 2.01 (1.70-2.36) | 1.48 (1.20-1.82) |  | 1.60 (1.24-2.06) | 1.39 (0.95-2.03) |  | 0.6758 |
|  |  |  |  |  |  |  |  |

HFrEF, heart failure with reduced e ejection fraction; HFpEF, heart failure with preserved ejection fraction; NYHA, New York Heart Association; LVEF, left ventricular ejection fraction; BMI, body mass index; ACEi, angiotensin converting enzyme inhibitors; ARB, angiotensin receptor blockers; MRA, mineralocorticoid receptor antagonist

| **Supplemental Table 3: Cardiovascular death or heart failure hospitalisation in HFrEF and HFpEF** | | | | | | | | |
| --- | --- | --- | --- | --- | --- | --- | --- | --- |
|  | HFrEF | | | | HFpEF | | | |
| Comorbidity group | No. at risk | No. of events (%) | Unadjusted  HR (95% CI) | Adjusted*  HR (95% CI) | No. at  risk | No. of events (%) | Unadjusted  HR (95% CI) | Adjusted*  HR (95% CI) |
| No DM, no CKD | 1163 | 143 (12.3%) | 1.00 (Ref) | 1.00 (Ref) | 367 | 16 (4.4%) | 1.00 (Ref) | 1.00 (Ref) |
| DM-only | 771 | 158 (20.5%) | 1.74 (1.39-2.18) | 1.51 (1.19-1.90) | 261 | 19 (7.3%) | 1.69 (0.87-3.28) | 1.23 (0.61-2.45) |
| CKD-only | 735 | 158 (21.5%) | 1.85 (1.48-2.32) | 1.47 (1.16-1.87) | 248 | 38 (15.3%) | 3.81 (2.13-6.84) | 2.77 (1.52-5.06) |
| DM+CKD | 944 | 320 (33.9%) | 3.15 (2.59-3.84) | 2.15 (1.73-2.68) | 378 | 75 (19.8%) | 5.02 (2.93-8.62) | 2.54 (1.43-4.53) |
| * Adjusted for age, sex, ethnicity, enrolment type, regional income, systolic blood pressure, heart rate, ejection fraction, coronary artery disease, chronic obstructive pulmonary disease, atrial fibrillation, peripheral arterial vascular disease, use of angiotensin converting enzyme inhibitors, angiotensin receptor blockers, beta blockers and diuretics.  † Adjusted for variables above* and education.  DM, Type 2 Diabetes; CKD, chronic kidney disease; HFrEF, heart failure with reduced ejection fraction; HFpEF, heart failure with preserved ejection fraction; | | | | | | | | |

| **Supplemental Table 4: Outcomes and HRQoL in HFrEF and HFpEF, by DM and CKD severity** | | | | | | | | | | | | | | | | |  |
| --- | --- | --- | --- | --- | --- | --- | --- | --- | --- | --- | --- | --- | --- | --- | --- | --- | --- |
| Composite outcome (death or HF hospitalization) at 1 year | | | | | | | | | | | | | | | | |  |
|  | **HFrEF** | | | | | | |  | **HFpEF** | | | | | | | |  |
| Comorbidity group | **No. at risk** | **No. of events (%)** | | **Crude HR (95% CI)** | | **Adjusted* HR (95% CI)** | |  | **No. at risk** | **No. of events (%)** | | **Crude HR (95% CI)** | | | **Adjusted* HR (95% CI)** | |  |
| No DM, no CKD | 1163 | 155 (13.3%) | | 1.00 (Ref) | | 1.00 (Ref) | |  | 367 | 20 (5.5%) | | 1.00 (Ref) | | | 1.00 (Ref) | |  |
| No DM, CKD-3a | 374 | 76 (20.3%) | | 1.57 (1.19-2.06) | | 1.25 (0.95-1.66) | |  | 131 | 17 (13.0%) | | 2.49 (1.31-4.76) | | | 1.91 (1.00-3.68) | |  |
| No DM, CKD-3b | 218 | 50 (22.9%) | | 1.81 (1.32-2.49) | | 1.47 (1.06-2.04) | |  | 72 | 13 (18.1%) | | 3.79 (1.89-7.63) | | | 2.61 (1.28-5.31) | |  |
| No DM, CKD-4 | 112 | 39 (34.8%) | | 3.14 (2.21-4.46) | | 2.37 (1.65-3.39) | |  | 28 | 13 (46.4%) | | 10.15 (5.05-20.4) | | | 5.65 (2.77-11.51) | |  |
| No DM, CKD-5 | 31 | 5 (16.1%) | | 1.26 (0.52-3.06) | | 1.09 (0.45-2.66) | |  | 17 | 2 (11.8%) | | 2.20 (0.51-9.42) | | | 1.26 (0.29-5.45) | |  |
| DM only, no CKD | 771 | 163 (21.1%) | | 1.66 (1.33-2.07) | | 1.44 (1.16-1.80) | |  | 261 | 19 (7.3%) | | 1.35 (0.72-2.53) | | | 1.18 (0.63-2.22) | |  |
| DM, CKD-3a | 378 | 102 (27.0%) | | 2.20 (1.72-2.83) | | 1.59 (1.23-2.06) | |  | 100 | 17 (17.0%) | | 3.33 (1.74-6.35) | | | 2.31 (1.20-4.46) | |  |
| DM, CKD-3b | 313 | 128 (40.9%) | | 3.57 (2.82-4.51) | | 2.46 (1.93-3.13) | |  | 143 | 32 (22.4%) | | 4.50 (2.57-7.86) | | | 3.07 (1.74-5.40) | |  |
| DM, CKD-4 | 187 | 81 (43.3%) | | 4.04 (3.09-5.29) | | 2.72 (2.06-3.59) | |  | 93 | 27 (29.0%) | | 6.32 (3.55-11.27) | | | 3.25 (1.79-5.92) | |  |
| DM, CKD-5 | 66 | 27 (40.9%) | | 3.61 (2.40-5.44) | | 2.50 (1.65-3.78) | |  | 42 | 11 (26.2%) | | 5.12 (2.45-10.69) | | | 3.29 (1.56-6.94) | |  |
| * Adjusted for age, ethnicity, inpatient enrolment *(limited adjustment due to small numbers in some groups)* | | | | | | | | | | | | | | | | |  |
| KCCQ at baseline | | | | | | | | | | | | | | | | |  |
|  | **Unadjusted** | | | | **Adjusted*** | | |  | **Unadjusted** | | | | **Adjusted*** | | | |  |
|  | **Mean (SEM)** | | **P-value** | | **Mean (SEM)** | | **P-value** |  | **Mean (SEM)** | | **P-value** | | | **Mean (SEM)** | | **P-value** | |
| No DM, no CKD | 67.2 (0.71) | | Ref | | 66.0 (0.65) | | Ref |  | 78.9 (1.24) | | Ref | | | 75.9 (0.99) | | Ref | |
| No DM, CKD-3a | 63.0 (1.25) | | 0.003 | | 62.6 (1.11) | | 0.008 |  | 74.5 (2.26) | | 0.096 | | | 74.3 (1.74) | | 0.424 | |
| No DM, CKD-3b | 61.5 (1.61) | | 0.001 | | 61.8 (1.42) | | 0.008 |  | 65.8 (2.91) | | <0.001 | | | 71.2 (2.24) | | 0.058 | |
| No DM, CKD-4 | 56.2 (2.19) | | <0.001 | | 58.4 (1.93) | | <0.001 |  | 60.2 (4.44) | | <0.001 | | | 67.0 (3.33) | | 0.012 | |
| No DM, CKD-5 | 59.3 (3.76) | | 0.038 | | 61.5 (3.35) | | 0.189 |  | 58.2 (5.73) | | <0.001 | | | 64.8 (4.81) | | 0.024 | |
| DM only, no CKD | 65.2 (0.90) | | 0.08 | | 65.3 (0.80) | | 0.488 |  | 74.8 (1.47) | | 0.034 | | | 72.7 (1.12) | | 0.03 | |
| DM, CKD-3a | 58.7 (1.35) | | <0.001 | | 59.1 (1.19) | | <0.001 |  | 69.2 (2.53) | | 0.001 | | | 70.1 (1.90) | | 0.007 | |
| DM, CKD-3b | 61.8 (1.45) | | 0.001 | | 63.2 (1.29) | | 0.055 |  | 69.8 (2.28) | | 0.001 | | | 71.6 (1.78) | | 0.039 | |
| DM, CKD-4 | 55.9 (1.92) | | <0.001 | | 58.8 (1.72) | | <0.001 |  | 62.2 (2.61) | | <0.001 | | | 70.1 (2.04) | | 0.013 | |
| DM, CKD-5 | 58.9 (2.89) | | 0.005 | | 59.3 (2.58) | | 0.013 |  | 59.0 (3.80) | | <0.001 | | | 64.2 (3.10) | | <0.001 | |
| * Adjusted for age, sex, ethnicity, enrolment type, regional income, systolic blood pressure, heart rate, ejection fraction, CAD, COPD, AF, PAVD, education, use of ACE-inhibitor/ARB, beta-blocker or diuretic | | | | | | | | | | | | | | | | |  |

DM, Diabetes mellitus; CKD, chronic kidney disease; HFrEF, heart failure with reduced ejection fraction; HFpEF, heart failure with preserved ejection fraction**;** SEM, standard error of mean

| **Supplemental Table 5. Outcomes and HRQoL in HFrEF and HFpEF (excluding Type 1 DM)** | | | | | | | | | | | | | | | |
| --- | --- | --- | --- | --- | --- | --- | --- | --- | --- | --- | --- | --- | --- | --- | --- |
|  | HFrEF | | | | | | | | HFpEF | | | | | | |
| Comorbidity group | **No. at risk** | | **No. of events (%)** | | **Unadjusted**  **HR (95% CI)** | | **Adjusted***  **HR (95% CI)** | | **No. at risk** | **No. of events (%)** | | **Unadjusted**  **HR (95% CI)** | | **Adjusted***  **HR (95% CI)** | |
| All-cause deaths or heart failure hospitalization at 1 year | | | | | | | | | | | | | | | |
| No T2D, no CKD | 1163 | | 155 (13.3%) | | 1.00 (Ref) | | 1.00 (Ref) | | 367 | 20 (5.5%) | | 1.00 (Ref) | | 1.00 (Ref) | |
| T2D-only | 645 | | 115 (17.8%) | | 1.36 (1.07-1.73) | | 1.32 (1.03-1.69) | | 220 | 10 (4.6%) | | 0.83 (0.39-1.76) | | 0.70 (0.31-1.56) | |
| CKD-only | 735 | | 170 (23.1%) | | 1.84 (1.48-2.29) | | 1.40 (1.11-1.77) | | 248 | 45 (18.2%) | | 3.62 (2.14-6.13) | | 2.50 (1.43-4.36) | |
| DM+CKD | 764 | | 254 (33.3%) | | 2.79 (2.28-3.40) | | 2.01 (1.61-2.51) | | 295 | 58 (19.7%) | | 3.87 (2.33-6.43) | | 2.05 (1.18-3.58) | |
| All-cause deaths or all-cause hospitalization at 1 year | | | | | | | | | | | | | | | |
| No T2D, no CKD | 1163 | | 302 (26.0%) | | 1.00 (Ref) | | 1.00 (Ref) | | 367 | 56 (15.3%) | | 1.00 (Ref) | | 1.00 (Ref) | |
| T2D-only | 645 | | 186 (28.9%) | | 1.11 (0.92-1.33) | | 1.09 (0.91-1.32) | | 220 | 36 (16.4%) | | 1.07 (0.70-1.62) | | 0.90 (0.58-1.40) | |
| CKD-only | 735 | | 261 (35.6%) | | 1.44 (1.22-1.70) | | 1.16 (0.97-1.38) | | 248 | 79 (31.9%) | | 2.33 (1.66-3.29) | | 1.67 (1.16-2.41) | |
| DM+CKD | 764 | | 343 (45.0%) | | 1.94 (1.66-2.27) | | 1.53 (1.28-1.81) | | 295 | 106 (35.9%) | | 2.71 (1.96-3.75) | | 1.76 (1.23-2.51) | |
| KCCQ at baseline | | | | | | | | | | | | | | | |
|  | **Unadjusted** | | | | | **Adjusted†** | | | **Unadjusted** | | | **Adjusted†** | | | |
|  | | **Mean (SEM)** | | **P-value** | | **Mean (SEM)** | | **P-value** | **Mean (SEM)** | **P** | **Mean (SEM)** | | **P** | |  |
| No T2D, no CKD | | 67.23 (0.71) | | Ref | | 66.01 (0.65) | | Ref | 78.85 (1.26) | Ref | 75.78 (0.99) | | Ref | |  |
| T2D-only | | 65.11 (0.91) | | 0.066 | | 65.18 (0.80) | | 0.423 | 74.64 (1.50) | 0.031 | 72.65 (1.13) | | 0.037 | |  |
| CKD-only | | 61.25 (0.88) | | <0.001 | | 61.69 (0.79) | | <0.001 | 68.81 (1.61) | <0.001 | 71.74 (1.24) | | 0.013 | |  |
| DM+CKD | | 59.32 (0.85) | | <0.001 | | 60.54 (0.78) | | <0.001 | 66.30 (1.37) | <0.001 | 70.20 (1.07) | | <0.001 | |  |
| * Adjusted for age, sex, ethnicity, enrolment type, regional income, systolic blood pressure, heart rate, ejection fraction, coronary artery disease, chronic obstructive pulmonary disease, atrial fibrillation, peripheral arterial vascular disease, use of angiotensin converting enzyme inhibitors, angiotensin receptor blockers, beta blockers and diuretics.  † Adjusted for variables above* and education.  T2D, Type 2 Diabetes; CKD, chronic kidney disease; HFrEF, heart failure with reduced ejection fraction; HFpEF, heart failure with preserved ejection fraction; SEM, standard error of mean. | | | | | | | | | | | | | | | |

## Additional references 41-52

## 41. Kaur-Gill S, Dutta MJ, Bashir MB. A Community-Based Heart Health Intervention: Culture-Centered Study of Low-Income Malays and Heart Health Practices. Frontiers in Communication. 2020;5:16.

## 42. Benes J, Kotrc M, Jarolim P, Hoskova L, Hegarova M, Dorazilova Z, et al. The effect of three major co-morbidities on quality of life and outcome of patients with heart failure with reduced ejection fraction. ESC Heart Fail. 2021 Apr;8(2):1417-26.

## 43. Lawson CA, Solis-Trapala I, Dahlstrom U, Mamas M, Jaarsma T, Kadam UT, et al. Comorbidity health pathways in heart failure patients: A sequences-of-regressions analysis using cross-sectional data from 10,575 patients in the Swedish Heart Failure Registry. PLoS Med. 2018 Mar 27;15(3):e1002540.

## 44. Burns DJP, Arora J, Okunade O, Beltrame JF, Bernardez-Pereira S, Crespo-Leiro MG, et al. International Consortium for Health Outcomes Measurement (ICHOM): Standardized Patient-Centered Outcomes Measurement Set for Heart Failure Patients. JACC: Heart Failure. 2020;8(3):212-22.

## 45. Spertus JA. Quality of life in EMPEROR-Reduced: emphasizing what is important to patients while identifying strategies to support more patient-centred care. Eur Heart J. 2021;42(13):1213-5.

## 46. Maddox Thomas M, Januzzi James L, Allen Larry A, Khadijah B, Javed B, Davis Leslie L, et al. 2021 Update to the 2017 ACC Expert Consensus Decision Pathway for Optimization of Heart Failure Treatment: Answers to 10 Pivotal Issues About Heart Failure With Reduced Ejection Fraction. J Am Coll Cardiol. 2021;77(6):772-810.

## 47. Clare A, Li Jing-Wei, Cannon Christopher P, Brendon N, Heerspink Hiddo L, Bruce N, et al. The effects of Canagliflozin on heart failure and cardiovascular death by baseline participant characteristics: analysis of the credence trial. J Am Coll Cardiol. 2020;75(11):674.

## 48. Pitt B, Filippatos G, Agarwal R, Anker SD, Bakris GL, Rossing P, et al. Cardiovascular Events with Finerenone in Kidney Disease and Type 2 Diabetes. N Engl J Med. 2021.

## 49. Wheeler DC, Stefánsson BV, Jongs N, Chertow GM, Greene T, Hou FF, et al. Effects of dapagliflozin on major adverse kidney and cardiovascular events in patients with diabetic and non-diabetic chronic kidney disease: a prespecified analysis from the DAPA-CKD trial. The Lancet Diabetes & Endocrinology. 2021;9(1):22-31.

## 50. McMurray JJV, Solomon SD, Docherty KF, Jhund PS. The Dapagliflozin and Prevention of Adverse outcomes in Heart Failure trial (DAPA-HF) in context. Eur Heart J. 2021 Mar 31;42(13):1199-202.

## 51. Solomon SD, Vaduganathan M, L Claggett B, Packer M, Zile M, Swedberg K, et al. Sacubitril/Valsartan Across the Spectrum of Ejection Fraction in Heart Failure. Circulation. 2020 Feb 4;141(5):352-61.

## 52. Lam CS, Teng TK, Tay WT, Anand I, Zhang S, Shimizu W, et al. Regional and ethnic differences among patients with heart failure in Asia: the Asian sudden cardiac death in heart failure registry. Eur Heart J. 2016;37(41):3141-53.

## The ASIAN-HF Executive Committee

• Professor A. Mark Richards (as Chairman), Cardiovascular Research Institute, National University of Singapore, Singapore. Email: mdcarthu@nus.edu.sg

• Professor Carolyn S.P. Lam (as Principal Investigator), National Heart Centre Singapore, Duke-NUS Medical School, Singapore. Email: carolyn.lam@duke-nus.edu.sg

• Professor Inder Anand (as Director, Publications Committee), University of Minnesota Medical School, VA Medical Center Minneapolis and San Diego, United States of America. Email: anand001@umn.edu

• Dr Chung-Lieh Hung, Mackay Memorial Hospital, Taipei, Taiwan. Email: jotaro3791@gmail.com

• Professor Lieng Hsi Ling (as Director, Echo Core Laboratory), Cardiovascular Research Institute, National University of Singapore, Singapore. Email: lieng_hsi_ling@nuhs.edu.sg

• Dr Houng Bang Liew, Queen Elizabeth II Hospital, Clinical Research Center, Sabah, Malaysia. Email: hbliew22@gmail.com

• Dr Calambur Narasimhan, Care Hospital, Hyderabad, India. Email: calambur@hotmail.com

• Dr Tachapong Ngarmukos, Ramathibodi Hospital, Mahidol University, Bangkok, Thailand. Email: tachaponis.nga@mahidol.ac.th

• Dr Sang Weon Park, SeJong General Hospital, Seoul, South Korea. Email: swparkmd@gmail.com

• Dr Eugenio Reyes, Manila Doctors Hospital, Manila, Philippines. Email: eugenereyes@yahoo.com

• Professor Bambang B. Siswanto, National Cardiovascular Center Universitas Indonesia, Jakarta, Indonesia. Email: bambbs@gmail.com

• Professor Wataru Shimizu, Department of Cardiovascular Medicine, Nippon Medical School, Tokyo, Japan. Email: wshimizu@nms.ac.jp

• Professor Shu Zhang, Fuwai Cardiovascular Hospital, Beijing, People’s Republic of China. Email: zsfuwai@vip.163.com

**COUNTRY AND SITE INVESTIGATORS**

**Hong Kong**

The Chinese University of Hong Kong: Cheuk Man Yu (Country PI).

**India**

CARE Hospital: Calambur Narasimhan (Country PI), B K S Sastry, Arun Gopi, K Raghu, C Sridevi, Daljeet Kaur. Care Institute of Medical Sciences: Ajay Naik, Keyur Parikh, Anish Chandarana, Urmil Shah, Milan Chag, Hemang Baxi, Satya Gupta, Jyoti Bhatia, Vaishali Khakhkhar, Vineet Sankhla, Tejas Patel, Vipul Kapoor. Hero Dayanand Medical College Heart Institute: Gurpreet Singh Wander, Rohit Tandon. Medanta-The Medicity: Vijay Chopra, Manoj Kumar, Hatinder Jeet Singh Sethi, Rashmi Verma, Sanjay Mittal. Sir Ganga Ram Hospital: Jitendra Sawhney, Manish Kr. Sharma. Westfort Hi-Tech Hospital Ltd: Mohanan Padinhare Purayil.

**Indonesia**

Rumah Sakit Jantung dan Pembuluh Darah Harapan Kita: Bambang Budi Siswanto (Country PI). RS Dr Hasan Sadikin: Pintoko Tedjokusumo, Erwan Martanto, Erwinanto. R S Khusus Jantung Binawaluya: Muhammad Munawar, Jimmy Agung Pambudi. RS Siloam Karawaci: Antonia Lukito, Ingrid Pardede, Alvin Thengker, Vito Damay, Siska Suridanda Danny, Rarsari Surarso.

**Japan**

Nippon Medical School: Wataru Shimizu (Country PI), National Cerebral and Cardiovascular Center: Takashi Noda, Ikutaro Nakajima, Mitsuru Wada, Kohei Ishibashi. Kinki University Hospital Cardiovascular Center: Takashi Kurita, Ryoubun Yasuoka. Nippon Medical School Hospital: Kuniya Asai, Kohji Murai, Yoshiaki Kubota, Yuki Izumi.Toho University Omori Medical Center: Takanori Ikeda, Shinji Hisatake, Takayuki Kabuki, Shunsuke Kiuchi, Tokyo Women's Medical University: Nobuhisa Hagiwara, Atsushi Suzuki, Dr. Tsuyoshi Suzuki.

Korea

SeJong General Hospital: Sang-Weon Park (Country PI), Suk Keun Hong, SookJin Lee, Lim Dal Soo, Dong-Hyeok Kim. Korea University Anam Hospital: Jaemin Shim, Seong-Mi Park, Seung-Young Roh, Young Hoon Kim, Mina Kim, Jong-Il Choi. Korea University Guro Hospital: Jin Oh Na, Seung Woon Rha, Hong Seog Seo, Dong Joo Oh, Chang Gyu Park, Eung Ju Kim, Sunki Lee,

Severance Hospital, Yonsei University Health System: Boyoung Joung, Jae-Sun Uhm, Moon Hyoung Lee, In-Jeong Cho, Hui-Nam Park. Chonnam National University Hospital: Hyung-Wook Park, Jeong-Gwan Cho, Namsik Yoon, KiHong Lee, Kye Hun Kim. Korea University Ansan Hospital: Seong Hwan Kim.

Malaysia

Hospital Queen Elizabeth II: Houng Bang Liew (Country PI), Sahrin Saharudin, Boon Cong Beh, Yu Wei Lee, Chia How Yen, Mohd Khairi Othman, Amie-Anne Augustine, Mohd Hariz Mohd Asnawi, Roberto Angelo Mojolou, You Zhuan Tan, Aida Nurbaini Arbain, Chii Koh Wong. Institut Jantung Negara: Razali Omar, Azmee Mohd Ghazi, Surinder Kaur Khelae, David S.P. Chew, Lok Bin Yap, Azlan Hussin, Zulkeflee Muhammad, Mohd. Ghazi Azmee. University Malaya Medical Centre: Imran Zainal Abidin, Ahmad Syadi Bin Mahmood Zhudi, Nor Ashikin Md Sari, Ganiga Srinivasaiah Sridhar, Ahmad Syadi Mahmood Zuhdi. Muhammad Dzafir Ismail. Sarawak General Hospital Heart Centre: Tiong Kiam Ong, Yee Ling Cham, Ning Zan Khiew, Asri Bin Said, Alan Yean Yip Fong, Nor Hanim Mohd Amin, Keong Chua Seng, Sian Kong Tan, Kuan Leong Yew.

Philippines

Manila Doctors Hospital: Eugenio Reyes (Country PI), Jones Santos, Allan Lim. Makati Medical Center: Raul Lapitan, Ryan Andal, Philippine Heart Center: Eleanor Lopez.

Singapore

National Heart Centre Singapore: Carolyn S.P. Lam (Country PI), Kheng Leng David Sim, Boon Yew Tan, Choon Pin Lim, Louis L.Y. Teo, Laura L.H. Chan. National University Heart Centre: Lieng Hsi Ling, Ping Chai, Ching Chiew Raymond Wong, Kian Keong Poh, Tan Tock Seng Hospital: Poh Shuan Daniel Yeo, Evelyn M. Lee, Seet Yong Loh, Min Er Ching, Deanna Z.L. Khoo, Min Sen Yew, Wenjie Huang. Changi General Hospital-Parent: Kui Toh Gerard Leong, Jia Hao Jason See, Yaozong Benji Lim, Svenszeat Tan, Colin Yeo, Siang Chew Chai. Singapore General Hospital-Parent: Fazlur Rehman Jaufeerally, Haresh Tulsidas, Than Aung. Khoo Teck Puat Hospital: Hean Yee Ong, Lee Fong Ling, Dinna Kar Nee Soon

Taiwan

Mackay Memorial Hospital, Taipei, Taiwan: Chung-Lieh Hung (Country PI), Hung-I Yeh,Jen-Yuan Kuo, Chih-Hsuan Yen. National Taiwan University Hospital: Juey-Jen Hwang, Kuo-Liong Chien, Ta-Chen Su, Lian-Yu Lin, Jyh-Ming Juang, Yen-Hung Lin, Fu-Tien Chiang, Jiunn-Lee Lin, Yi-Lwun Ho, Chii-Ming Lee, Po-Chih Lin, Chi-Sheng Hung, Sheng-Nan Chang, Jou-Wei Lin, Chih-Neng Hsu. Taipei Veterans General Hospital: Wen-Chung Yu, Tze-Fan Chao, Shih-Hsien Sung, Kang-Ling Wang, Hsin-Bang Leu, Yenn-Jiang Lin, Shih-Lin Chang, Po-Hsun Huang, Li-Wei Lo, Cheng-Hsueh Wu. China Medical University Hospital: Hsin-Yueh Liang, Shih-Sheng Chang, Lien-Cheng Hsiao, Yu-Chen Wang, Chiung-Ray Lu, Hung-Pin Wu, Yen-Nien Lin, Ke-Wei Chen, Ping-Han Lo, Chung-Ho Hsu, Li-Chuan Hsieh.

Thailand

Ramathibodi Hospital: Tachapong Ngarmukos (Country PI), Mann Chandavimol, Teerapat Yingchoncharoen, Prasart Laothavorn. Phramongkutklao Hospital:Waraporn Tiyanon. Maharaj Nakorn Chiang Mai Hospital: Wanwarang Wongcharoen, Arintaya Phrommintikul.
